# A Scientometric Visualization Analysis for Natural Products on Cancer Research from 2008 to 2020

**DOI:** 10.3389/fphar.2021.650141

**Published:** 2021-08-06

**Authors:** Haitao Chen, Rongrong Li, Fan Zhang, Qinghua Yao, Yong Guo

**Affiliations:** ^1^ The First Clinical College of Zhejiang Chinese Medical University, Hangzhou, China; ^2^ The Third Clinical College of Zhejiang Chinese Medical University, Hangzhou, China; ^3^ Department of Integrated Traditional Chinese and Western Medicine, The Cancer Hospital of the University of Chinese Academy of Sciences (Zhejiang Cancer Hospital), Hangzhou, China; ^4^ Department of Oncology, The First Affiliated Hospital of Zhejiang Chinese Medical University, Hangzhou, China

**Keywords:** natural product, cancer, bibliometric, citation analysis, molecular docking, gut microbiota, immune checkpoint

## Abstract

**Background:** An increasing number of studies have shown that natural products have anti-tumor effects, and it has become a hotspot in cancer research. However, few bibliometric analyses have been examined in this field systematically. The current study aimed to explore the status and provide the developing trends in the natural products on cancer research.

**Methods:** Publications on natural products in cancer research were extracted from the Web of Science core collection database. CiteSpace (5.6.R3) software and GraphPad prism 6 were used to analyze and plot the references.

**Results:** On February 1, 2021, 34,611 records of natural products in cancer research published from 2008 to 2020 were collected. The United States was the driving force, with a strong academic reputation in this area. The top-contributing institution was the Chinese Academy of Sciences. Most publications were published in *Molecules.* Efferth Thomas was the most prolific author, while Newman DJ was the most cited and frequently co-cited author. Flavonoid, curcumin, and polyphenol were the most widely studied natural products. Oleanolic acid and rosmarinic acid have gradually become research hotspots recently. Breast cancer, prostate cancer, and colorectal cancer were the most common types of cancer in this field. “Natural killer cell” was the leading research hotspot. The keywords of “leaf extract,” “molecular docking” and “gold nanoparticle” appeared most recently as research frontiers.

**Conclusion:** Our results provided a general overview of the major research directions of natural products research in cancer. The mechanisms of natural products, especially those related to molecular docking, gold nanoparticle, gut microbiota, and immune checkpoints may soon become hotspots and should be closely monitored.

## Introduction

The incidence of cancer is rapidly growing worldwide. The World Health Organization (WHO) reported that cancer was the second leading cause of death globally. It was estimated to account for 18.1 million new cancer cases and 9.6 million deaths in 2018 (Bray et al., 2018). Although cancer mortality has decreased currently, especially in developed countries (Siegel et al., 2020), the increasing incidence of cancer still brings a heavy burden to the globe. Moreover, cancer also brings a financial burden to patients and affects their health-related quality of life (Koskinen et al., 2019). At present, surgery, chemo-radiotherapy, targeted therapy, and immunotherapy are the main clinical therapies for cancer treatment (Schnipper et al., 2015). However, these therapies can lead to adverse reactions in patients (Martins et al., 2019; Naveed et al., 2019; Deng et al., 2020), such as diarrhea, cardiotoxicity, and dermatitis, etc. Therefore, finding optimally acting drugs with lower side effects to treat it is warranted.

Increasing evidence to implicate natural products have the effect of anti-tumor in different cancers. Several reviews have summarized the research of common natural products in cancer. For instance, berberine (extract from *Coptis chinensis*, *Cortex Phellode* and *Berberis*) (Hallajzadeh et al., 2020), curcumin (extracted from the *Curcuma longa plant*) (Shafabakhsh et al., 2019), erianin (extracted from *Dendrobium chrysotoxum Lindl*) (Zhang et al., 2019) were commonly used drugs in the anti-cancer study. The mechanisms of anti-tumor action include the promotion of apoptosis (Shahwar et al., 2019), inhibition of inflammatory response (Qin et al., 2017), regulation of oxidative stress (Rosado-Pérez et al., 2019), modulation of gut microbiota (Meng et al., 2018), and improvement of immunity (Chen and Yu, 2016).

Notably, several natural products have been used worldwide for inhibiting tumors in patients with cancer. For example, paclitaxel, isolated from taxaceae, is one of the commonly used chemotherapy drugs for breast cancer (Yamamoto et al., 2017) and ovarian cancer (Blagden et al., 2020), improving patients’ survival time effectively. Additionally, Fang et al. conducted a double-blind, randomized, placebo-controlled clinical trial and confirmed that berberine was safe and effective in reducing the risk of recurrence of colorectal adenoma (Chen et al., 2020a). However, it should be noted that many pieces of evidence for anti-cancer action of natural products came from *in vitro* and *in vivo* preclinical studies, and only a few natural products have been used in clinic.

With the rapid development of natural products in cancer research, it is essential to identify the most productive contributors, the key topics, and highly frequent keywords, and to grasp abreast of emerging trends in the development of relevant knowledge. However, there are few systematic analyses of these publications. Bibliometric analysis has been widely used to evaluate the literature quantitatively and explore developmental trends in many research fields (Stout et al., 2018; Mulet-Forteza et al., 2019). To our knowledge, only Yeung et al. (2018) conducted a literature analysisof 8,012 articles on the molecular responses of cancers by natural products in 2018. However, no comprehensive systematic review and bibliometric analysis of natural products in cancer research have been performed. Therefore, to better understand the current situation and trends of natural products on cancer research, the purpose of this study was to visualize the references with vivid information by using bibliometric methods, reveal the current research trends, and explore the potential hotspots for researchers and guide their future work.

## Methods

### Data Source

The Web of Science core collection online database was queried with the following search string: TS = (“natural product*“ OR “natural compound*” OR “natural molecule*” OR “phytochemical*” OR “secondary metabolite*”) AND (“cancer*” OR “neoplasm*” OR “tumor*”). Only original articles and reviews written in English and published from 2008 to 2020 were included.

### Data Collection and Analysis

All records were downloaded by two authors (HT Chen and RR Li) independently from the Web of Science core collection, including the number of annual publications, outputs of countries/regions, institutions, journals, total citation, citations per publication (CPP) and Hirsch index (H-index). The impact factor (IF) and quartile of a journal category were obtained from Journal Citation Reports (JCR) 2019. Any disagreements were resolved by consensus.

We used the GraphPad prism 6 to analyze and plot annual publication output. Besides, the current research basis, cutting-edge knowledge and research trends were obtained by using bibliometric methodology (Merigó et al., 2019). In this study, all the download data were converted to the CiteSpace (5.6.R3) software, a visualization tool invented by Professor Chaomei Chen (Xu et al., 2017), which was used to analyze the basic metrics, including co-citation analysis of the countries, journals, institutions, authors, and references, as well as the timeline view of co-cited references. In addition, the CiteSpace software can capture keywords with strong citation bursts and construct visualization maps of all items. The parameters of CiteSpace were set as below: time-slicing was from 2008 to 2020, years per slice (1), and all options in the term source were selected, one node type was selected at a time, selection criteria (top 50 objects). Thus, the network map was extracted from the top 50 cited papers in 1 year per slice. Visualization knowledge figures consist of nodes and links. Each node in the figure represented an element, including country, institution, co-cited author, co-cited reference, and keyword. The size of node represented the frequency of appearance or citation, and the different colors of nodes indicated the different years. The circles of different colors from the inside to the outside of the node represented the year from 2008 to 2020. The nodes in the outermost area with a purple ring indicated high centrality, which was usually considered as pivotal points or key points in a specific field. Besides, lines between the nodes signified cooperation or co-occurrence or co-citation relationships.

## Results

### Annual Publications

**FIGURE 1:**
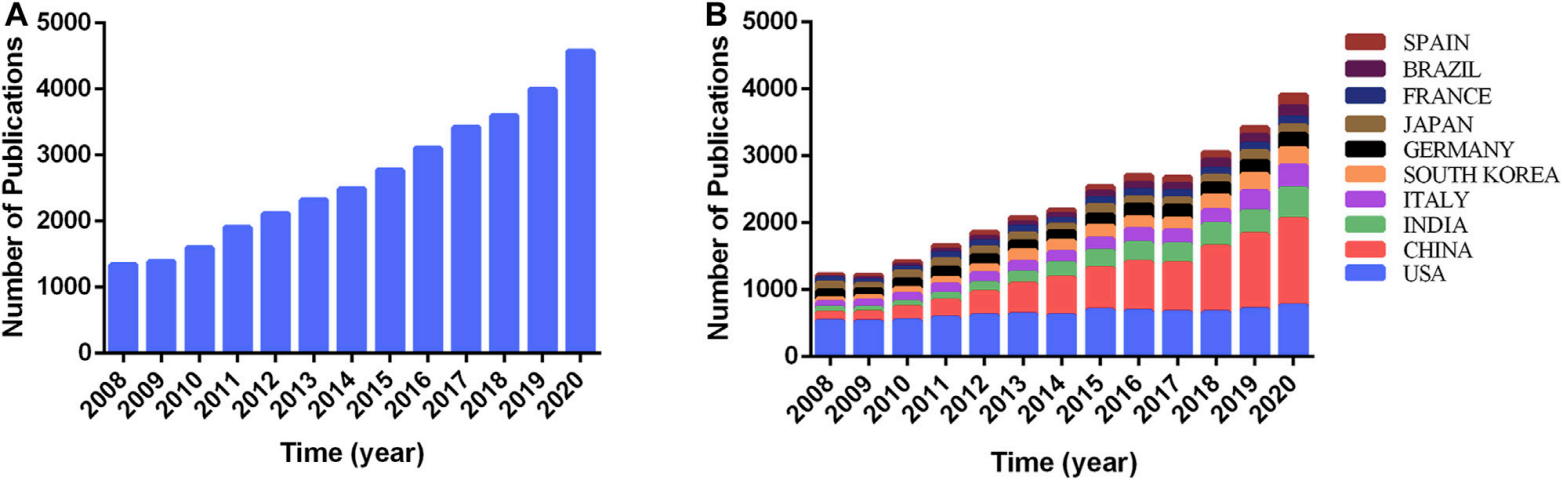
Trends in the number of publications of country/regions in natural products in cancer research. **(A)** The annual worldwide publication output. **(B)** The annual national publication output of the 10 most productive countries/regions.

From 2008 to 2020, a total of 34,611 publications were extracted, including 27,131 articles and 7,480 reviews. The growth of the annual publication output was shown in Figure 1A. The annual publication output increased annually, and increased to 4,000 in 2019 to nearly three times the annual publication output in 2008. Importantly, 4,573 related publications have been published in 2020 to date, although this count does not reflect the total number of publications during the whole year.

### Distribution of Countries/Regions and Institutions

All publications were distributed among 161 countries/regions and 793 institutions. The United States had the highest output with 8,029 papers (23.19% of 34,611 papers), followed by China (22.28%, with 7,711 papers), India (8.17%, with 2,826 papers), Italy (6.80%, with 2,355 papers), and South Korea (5.96%, with 2,064 papers) (Table 1). In addition, we further identified the annual national publication output of the 10 most productive countries/regions (Figure 1B). The United States has ranked first in the number of annual publications from 2008 to 2015, but China had more annual publications than that in the United States since 2016. Meanwhile, the annual growth rate of publication output increased the fastest in China since 2008, followed by Brazil and India. However, the annual publication output in Japan maintained a similar level from 2008 to 2020.

**TABLE 1:** The top 10 productive countries/regions related to natural products in cancer research.

Rank	Country/region	Article counts	Percentage (N/34611)	H (%)- index	Citations	Citations per publication
1	United States	8,029	23.19	194	289,719	36.08
2	China	7,711	22.28	107	132,635	17.20
3	India	2,826	8.17	92	55,826	19.75
4	Italy	2,355	6.80	98	62,195	26.41
5	South Korea	2,064	5.96	76	39,450	19.11
6	Germany	2,053	5.93	103	62,617	30.50
7	Japan	1,551	4.48	77	36,056	23.25
8	France	1,186	3.43	84	34,787	29.33
9	Brazil	1,123	3.24	54	17,611	15.68
10	Spain	1,114	3.22	80	30,103	27.02

In the 10 top countries/regions of publications, The United States had 289,719 citations and an H-index of 194, both of which ranked first among all included countries/regions, and its citation/publication ratio (36.08) was also the highest. Although China had relatively high citations (132,635) and H-index (107), its citation/publication ratio (17.20) was lower than other countries/regions, except for Brazil (Table 1).

**FIGURE 2:**
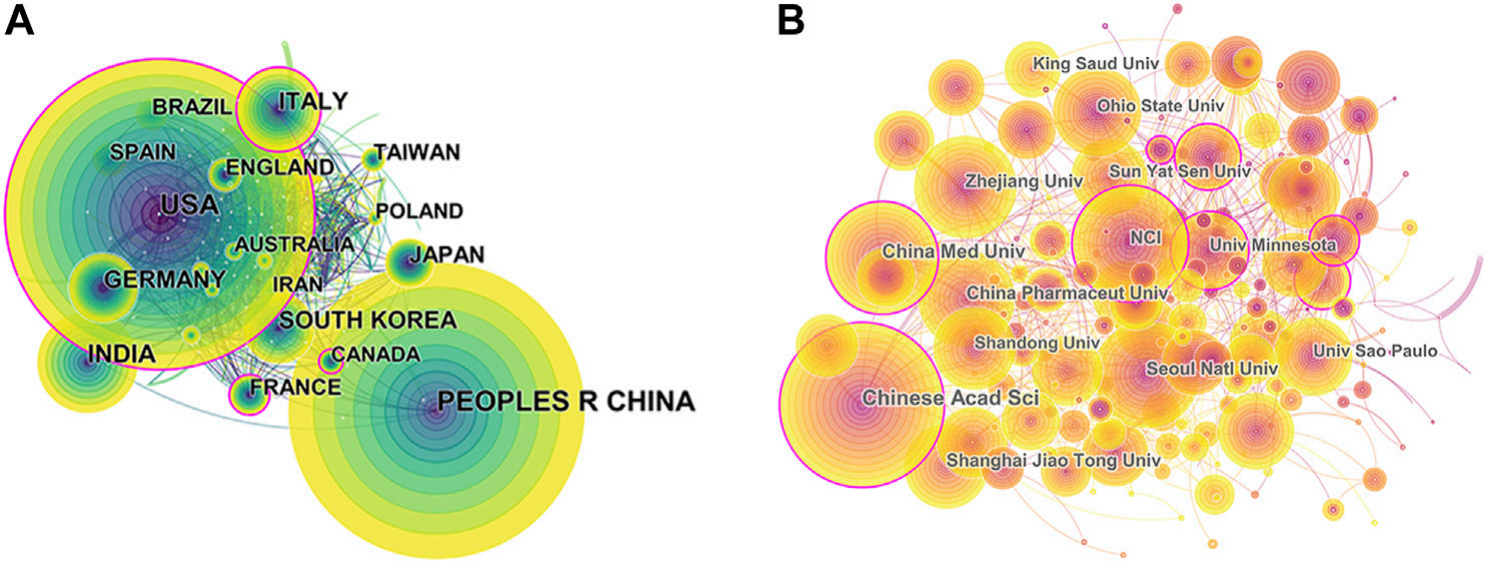
CiteSpace network visualization map of country/regions and institutions related to natural products in cancer research. **(A)** Collaboration analysis of countries/regions. **(B)** Collaboration analysis of institutions. The nodes represent countries/regions or institutions, and lines between the nodes represent cooperation relationships. The larger the node size, the larger the number of publications. The nodes in the outermost area with purple rings indicate high centrality.

International collaboration network analysis was shown in Figure 2A, demonstrating that the United States and China, with the largest output, cooperated closely. In addition, the countries/regions that collaborated most with the United States were Italy, Australia, Germany, Spain, Taiwan, South Korea, and India. The People’s Republic of China mainly cooperated with Canada, France, and Singapore.

The 10 most productive institutions were shown in Table 2. The leading institutions were the Chinese Academy of Sciences (2.74%, with 948 papers), University of California System (1.74%, with 601 papers), and Council of Scientific Industrial Research (1.59%, with 552 papers). More than half of the 10 most productive institutions came from the United States. The National Institutes of Health (United States) had the highest H-index (74), and its citation/publication ratio (55.13) was also the highest among the top 10 productive institutions. To uncover potential collaboration among institutions, a co-authorship analysis of institutions by CiteSpace was conducted (Figure 2B).

**TABLE 2:** The top 10 productive institutions related to natural products in cancer research.

Rank	Institution	Article counts	Percentage (N/34611)	H(%)-index	Citations per publication	Location
1	Chinese Academy of Sciences	948	2.74	66	22.27	China
2	University of California System	601	1.74	75	40.53	United States
3	Council of Scientific Industrial Research (CSIR)	552	1.59	47	18.75	India
4	Centre National De La Recherche Scientifique	551	1.59	59	26.47	France
5	National Institutes of Health (NIH)	497	1.44	74	55.13	United States
6	Institut National De La Sante Et De La Recherche Medicale (INSERM)	482	1.39	64	34.03	United States
7	University of Texas System	450	1.30	66	36.73	United States
8	Harvard University	359	1.04	60	42.28	United States
9	Helmholtz Association	355	1.03	53	30.01	Germany
10	Nih National Cancer Institute (NCI)	350	1.01	62	47.34	United States

### Analysis of Journals and Cited Journals

The 10 most productive and co-cited journals were listed in Tables 3, 4. *Molecules* (828 publications, 2.39%) published the most research in this field, which had an IF of 3.267 in 2019, followed by *Plos One* (615 publications, 1.78%), *International journal of molecular sciences* (449 publications, 1.30%), *Journal of natural products* (402 publications, 1.16%), and *Marine drugs* (401 publications, 1.16%). *European journal of medicinal chemistry* had the highest IF (5.572) in 2019 among the top 10 productive journals. *Molecules* had the highest H-index (55), and *Journal of natural products* had the highest citation/publication ratio (36.02) among the top 10 productive journals. Also, half of the productive journals were classified in Q1 (the top 25% of the IF distribution), and the left in Q2 (between the 25th percentile and 50th percentile).

**TABLE 3:** The top 10 productive journals related to natural products in cancer research.

Rank	Journal	Article counts	Percentage (N/34661)	I (%) F (2019)	H-index	Citations per article	Quartile in category
1	Molecules	828	2.39	3.267	55	18.98	Q2
2	Plos One	615	1.78	2.74	53	24.60	Q1
3	International Journal of Molecular Sciences	449	1.30	4.556	42	18.82	Q2
4	Journal of Natural Products	402	1.16	3.779	42	36.02	Q1
5	Marine Drugs	401	1.16	4.073	41	19.99	Q1
6	European Journal of Medicinal Chemistry	344	1.00	5.572	43	24.89	Q1
7	Bioorganic Medicinal Chemistry Letters	320	0.92	2.572	36	16.70	Q2
8	Phytochemistry Letters	314	0.91	1.459	22	9.95	Q2
9	Scientific Reports	309	0.89	3.998	32	14.73	Q1
10	Bioorganic Medicinal Chemistry	305	0.88	3.073	40	22.98	Q2

**TABLE 4:** The top 10 co-cited journals related to natural products in cancer research.

Rank	Cited Journal	Citations	IF (2019)	H-index	Quartile in category
1	Cancer Research	14,318	9.727	125	Q1
2	Proceedings of the National Academy of Sciences of the United States of America (PNAS)	13,037	9.412	—	Q1
3	Journal Of Biological Chemistry	12,764	4.238	92	Q2
4	Nature	11,536	42.779	368	Q1
5	Plos One	11,365	2.74	53	Q1
6	Science	10,462	41.846	338	Q1
7	Journal of Natural Products	8,858	3.782	43	Q1
8	Journal of Agricultural And Food Chemistry	8,475	4.192	71	Q1
9	Cancer Letters	8,470	7.36	85	Q1
10	Clinical Cancer Research	8,337	10.107	125	Q1

The most frequently co-cited journal in Q1 was *Cancer Research* (14,318 citations). The next most frequently co-cited journals were *Proceedings of the national academy of sciences of the United States of America* (13,037 citations), *Journal of biological chemistry* (12,764 citations). Among the top 10 co-cited journals, *Nature* had 11,536 citations, with the highest IF (42.779) and H-index (368). Most of the co-cited journals were in Q1.

### Analysis of Authors and Cited Authors

**FIGURE 3:**
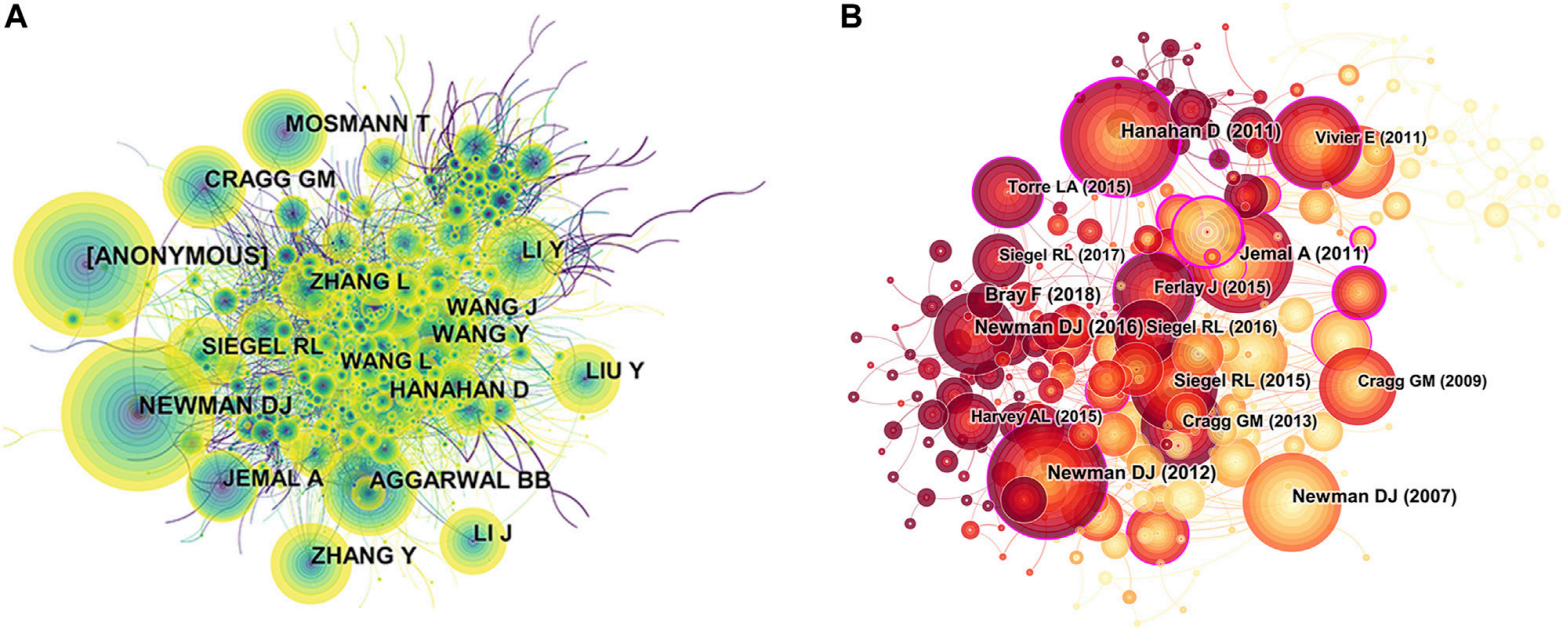
CiteSpace network visualization map of co-cited authors and co-cited references related to natural products in cancer research. **(A)** The network visualization map of co-cited authors of the publication. **(B)** The network visualization map of cited references. The nodes represent co-cited authors or cited references. The lines between the nodes represent co-citation relationships. The larger the node area, the larger the number of co-citations.

A total of 972 first authors published papers about the natural products in cancer study. The 10 most productive authors were shown in Table 5. Efferth Thomas (131 publications) published the most publications, followed by Wei Li (82 publications), Yan Li (73 publications), Wei Wang (72 publications), and Anupam Bishayee (58 publications). In addition, further analysis revealed that five authors were from China, and the remaining five were from the United States, Germany, South Korea, Belgium, and India, respectively. The network visualization map of the co-cited authors is shown in Figure 3A. The largest nodes were associated with the most frequently co-cited authors, including Newman DJ (2,245 citations), Aggarwal BB (1,441 citations), Wang Y (1,436 citations), Mosmann T (1,268 citations), and Cragg GM (1,242 citations) (Table 5).

**TABLE 5:** The top 10 productive authors and co-cited authors related to natural products in cancer research.

Rank	Author	Count	Location	Rank	Co-cited author	Citation
1	Efferth Thomas	131	Germany	1	Newman DJ	2,245
2	Wei Li	82	China	2	Aggarwal BB	1,441
3	Yan Li	73	China	3	Wang Y	1,436
4	Wei Wang	72	China	4	Mosmann T	1,268
5	Bishayee Anupam	58	United States	5	Cragg GM	1,242
6	Diederich Marc	49	South Korea	6	Zhang Y	1,236
7	Kiss Robert	44	Belgium	7	Li Y	1,218
8	Yu Zhang	44	China	8	Hanahan D	1,191
9	Jing Li	43	China	9	Siegel RL	1,082
10	Sahebkar Amirhossein	43	Iran	10	Wang J	1,032

### Analysis of Cited References

The network map of co-cited references consisted of 1,866 nodes and 2,937 links, with the time slice set as 1 year and the time span set as 2008 to 2020 (Figure 3B). According to the five most frequently co-cited references (Table 6), the title of “Hallmarks of cancer: the next generation” published in *Cell* (IF 2019, 38.637) was the most cited article, which was authored by Hanahan D, with 640 citations (Hanahan and Weinberg, 2011). Meanwhile, there were two co-cited references written by Newman DJ, which were published in *Journal of natural products* (IF 2019, 3.779) (Newman and Cragg, 2016; Newman and Cragg, 2012). Interestingly, the top most frequently co-cited reference also had the highest centrality. Besides, among the top five co-cited references ranked by centrality (Table 7), two articles were mainly related to the natural killer cell research (Lanier, 2008; Brandt et al., 2009). All of the top five ranked references by citation counts and centrality were in Q1.

**TABLE 6:** The top five high-cited references related to natural products in cancer research.

Rank	Title	Journal IF (2019)	First author	Publication time	Total citations	Quartile in category
1	Hallmarks of cancer: the next generation	Cell (IF: 38.637)	Hanahan D.	March 2011	640	Q1
2	Natural Products as Sources of New Drugs from 1981 to 2014	Journal of Natural Products (IF: 3.779)	Newman D. J.	March 2016	575	Q1
3	Natural products as sources of new drugs over the 30 years from 1981 to 2010	Journal of Natural Products (IF: 3.779)	Newman D. J.	March 2012	523	Q1
4	Global cancer statistics 2018: GLOBOCAN estimates of incidence and mortality worldwide for 36 cancers in 185 countries	CA-A Cancer Journal for Clinicians (IF: 292.278)	Bray F.	November 2018	422	Q1
5	Global cancer statistics	CA-A Cancer Journal for Clinicians (IF: 292.278)	Jemal A.	March 2011	405	Q1

**TABLE 7:** The top five centrality of co-cited references related to natural products in cancer research.

Rank	Title	Journal IF (2019)	First author	Publication time	Centrality	Quartile in category
1	Hallmarks of cancer: the next generation	Cell (IF: 38.637)	Hanahan D	March 2011	0.36	Q1
2	Cancer-related inflammation	Nature (IF: 42.778)	Mantovani A	July 2008	0.34	Q1
3	The B7 family member B7-H6 is a tumor cell ligand for the activating natural killer cell receptor NKp30 in humans	Journal Of Experimental Medicine (IF: 11.7434)	Brandt CS	July 2009	0.29	Q1
4	Molecular targets of phytochemicals for cancer prevention	Nature Reviews Cancer (IF: 53.03)	Lee KW	March 2011	0.22	Q1
5	Up on the tightrope: natural killer cell activation and inhibition	Nature Immunology (IF: 20.479)	Lanier LL	May 2008	0.18	Q1

**FIGURE 4:**
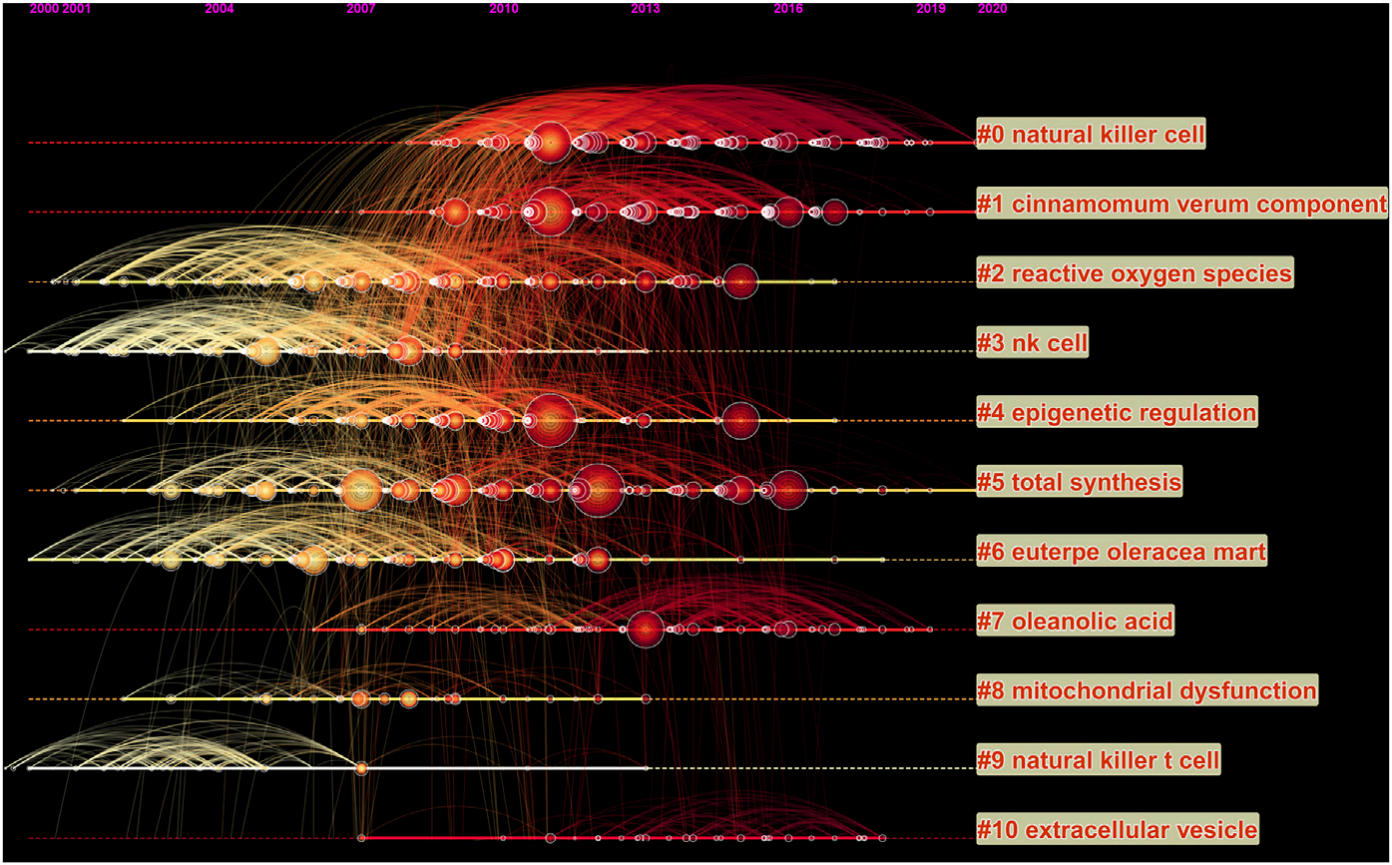
Timeline view of co-cited references related to natural products in cancer research. The cluster with warmer color and larger nodes contained more publications, indicating that this clustering issue was the hotspot in this field.

Furthermore, we performed a temporal co-citation analysis (Figure 4). Most articles were published after 2008. We found that “reactive oxygen species” (cluster #2) and “euterpe oleracea mart” (cluster #6) were relatively early research hotspots. Due to the difference of professional words used in the different periods, there were three categories of “natural killer cell,” including Cluster #0, Cluster #3, and Cluster #9, with the warmest color and largest nodes containing the most publications, indicating that this clustering issue is always the hotspot in natural products on cancer research during the past years. Interestingly, “cinnamomum verum component” (Cluster #1), “total synthesis” (Cluster #5), and “oleanolic acid” (Cluster #7) demonstrated that this clustering issue is the new hotspot and direction in this field currently.

### Analysis of Keywords and Keyword Co-Occurrence Clusters

According to the citation counts and centrality analysis of keywords by CiteSpace, we found that the most popular keywords were “apoptosis,” “cancer,” “natural product,” “*in vitro*,” “expression,” and “activation” (Table 8). Mechanism studies related to “cytotoxicity,” “NF-kappa B,” and apoptosis’ were most frequently investigated. Further analysis of the keywords showed that the natural products of “flavonoid,” “curcumin” and “polyphenol” were most frequently listed, and the cancer types of “breast cancer,” “prostate cancer,” and “colorectal cancer” were most frequently listed (Table 9).

**TABLE 8:** The top 10 frequency and centrality of keywords related to natural products in cancer research.

Rank	Keyword	Counts	Rank	Keyword	Centrality
1	Apoptosis	6,238	1	Apoptosis	0.35
2	Cancer	6,057	2	Expression	0.33
3	Natural product	4,565	3	Natural product	0.26
4	*In vitro*	4,041	4	*In vitro*	0.24
5	Expression	3,777	5	Cancer	0.16
6	Activation	2,868	6	Breast cancer	0.14
7	Cytotoxicity	2,351	7	NF kappa B	0.14
8	Breast cancer	2,146	8	Antioxidant	0.14
9	NF kappa B	2,130	9	Down regulation	0.13
10	Cell	2,123	10	Flavonoid	0.12

**TABLE 9:** The top five productive natural products and cancers related to natural products in cancer research.

Rank	Natural products or compound classes	Counts	Rank	Cancer	Counts
1	Flavonoid	1,327	1	Breast cancer	2,146
2	Curcumin	858	2	Prostate cancer	1,160
3	Polyphenol	564	3	Colorectal cancer	789
4	Resveratrol	501	4	Lung cancer	618
5	Phenolic Compound	100	5	liver cancer	103

**FIGURE 5:**
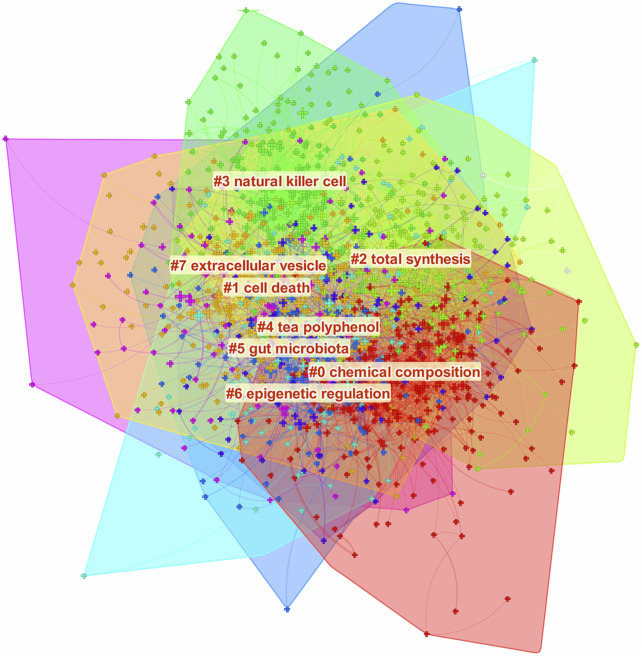
The cluster of keywords related to natural products in cancer research. The different colors mean different clusters.

Furthermore, we also constructed a network map to visualize the clusters of keywords (Figure 5). Cluster #0 labeling the “chemical composition,” was the largest cluster, followed by “cell death” (cluster #1), and “total synthesis” (cluster #2). Furthermore, several research directions, including “natural killer cell” (cluster #3), “gut microbiota” (cluster #5), and “epigenetic regulation” (cluster #6) were the main topics since 2008. Meanwhile, the natural product of “tea polyphenol” (cluster #4) indicated that it was also a research hotspot in this field.

### Analysis of Burst Keywords

**FIGURE 6:**
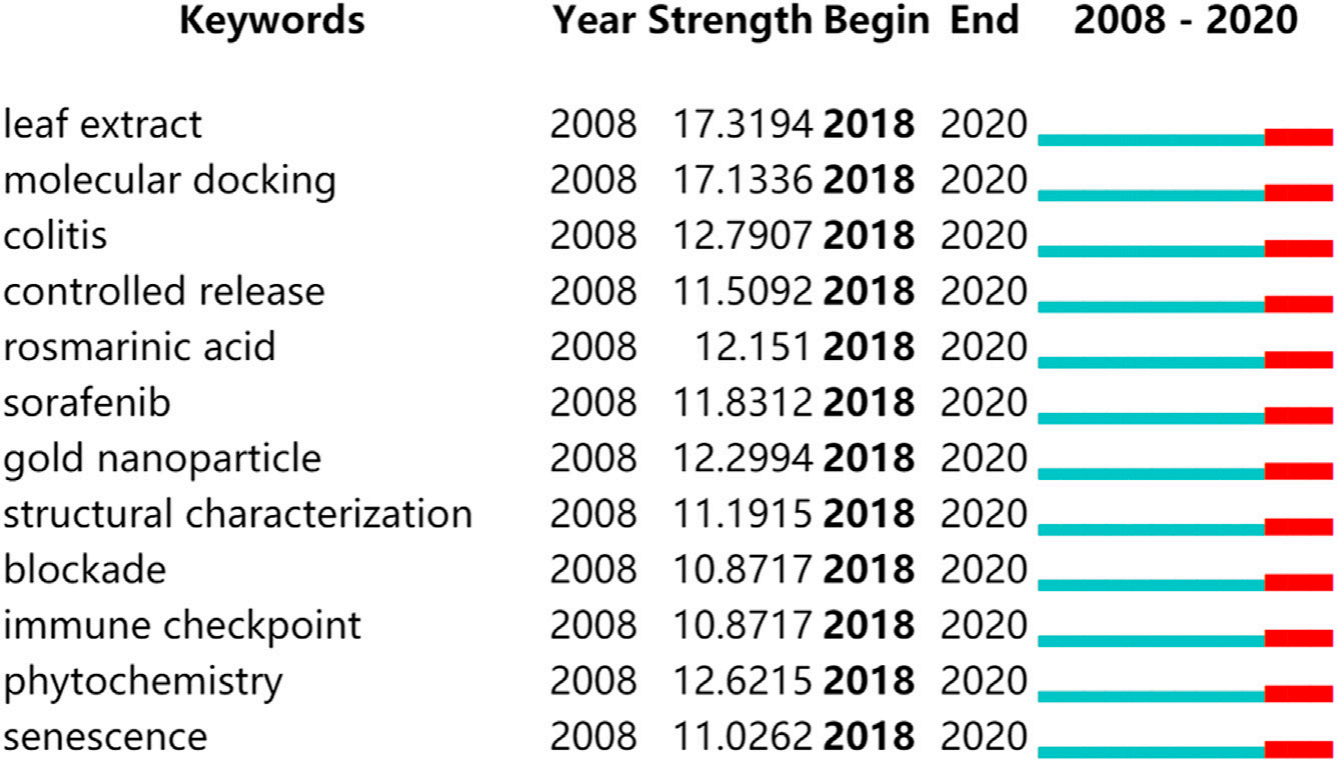
Keywords with periods of burst from 2018 onward among the top 75 burst keywords in articles related to natural products in cancer research.

We used CiteSpace software to detect burst keywords to determine the hotspots and research frontiers over time. Among the top 75 keywords with the strongest citation bursts in this field, we focused on those keywords that started to burst from 2018 onward (Figure 6), including “leaf extract” (with a burst strength of 17.3194), “molecular docking” (with a burst strength of 17.1336), “colitis” (with a burst strength of 12.7907), “controlled release” (with a burst strength of 11.5092), “rosmarinic acid” (with a burst strength of 12.151), “gold nanoparticle” (with a burst strength of 12.2994), “structural characterization” (with a burst strength of 11.1915), “phytochemistry” (with a burst strength of 12.6215), “senescence” (with a burst strength of 11.0262), and “immune checkpoint” (with a burst strength of 10.8717).

## Discussion

This study identified 34,611 publications related to natural products in cancer research through the Web of Science core collection database from 2008 to 2020. The annual publication output increased steadily. Referring to the distribution of countries/regions, the United States was the main driving force with a high academic reputation in natural products on cancer research, which was confirmed by the following characteristics: number of publications, H-index value, the total number of citations, and citations per publication (CPP). Recently, the annual publications in China increased significantly, and the number of annual publications surpassed that in the United States since 2016. Although publications in China had a low CPP, the H-index value and citation counts were relatively high, indicating that China also had a certain influence in this field. The total amount of research publications from other countries, such as Italy, Germany, Spain, and France, was relatively low. However, the recent increase in their annual output reflects the considerable progress made by these countries in this field, which was closely related to their strong collaborations with the United States.

Furthermore, among the top 10 productive institutions, more than half of the institutions came from the United States, showing the strong academic impact of the United States in this field. The Chinese Academy of Sciences was the most productive institution worldwide and collaborated tightly with many other Chinese agencies, indicating that it had a high academic reputation in this field in China. In addition, the National Institutes of Health (United States) had the highest H-index and CPP among institutions, which showed that it published more high-quality publications and played a critical role in promoting the development of this field.

### Journal, Cited Journal, Author, and Reference Analysis

Among the 10 most active journals, *Molecules* (55 publications) and *Plos One* (53 publications) had H- indeces higher than 50. In addition, *Plos One* and *Journal of natural products* were also among the leading co-cited journals, ranking fifth and seventh respectively, reflecting that all of them have been vital information resources. Furthermore, most literatures focused mainly on three categories, including pharmacology pharmacy, chemistry medicinal, and biochemistry molecular biology, indicating that these categories were highly recommended for tracking knowledge about natural products in cancer. Notably, the *Journal of natural products* mainly published articles about natural product research relating to the chemistry and/or biochemistry of naturally occurring compounds or the biology of living systems, with the highest CPP (36.02), which was one of the 10 most co-cited journals, a position related to contributions of two articles written by Newman DJ in 2012 (Newman and Cragg, 2012), and 2016 (Newman and Cragg, 2016), respectively. Therefore, this journal was recognized as a natural products research resource and had an essential influence on this research field. Interestingly, Newman D. J. from the National Institutes of Health (United States) ranked first in all co-cited authors. His two high-cited articles mainly elaborated on the clinical application of natural products as a source of new drugs to all diseases on a global scale. Thus, these articles have been regarded as reliable reference resources for later research. Additionally, the paper entitled “Hallmarks of cancer: the next generation” published in *Cell* was the highest frequency and centrality of co-cited reference, which mainly focused on summarizing the biological characteristics of tumors (Hanahan and Weinberg, 2011). Therefore, this article could provide insights for the study of the anti-tumor mechanism of natural products. Furthermore, according to the top five cited references ranked by centrality, two articles were mainly studied of natural killer cells (Lanier, 2008; Brandt et al., 2009). As we all know, natural killer cells are lymphocytes of the innate immune system that participate in the elimination of tumors. Therefore, the high citations of these two papers indicated the importance of natural killer cells in this field. The article of “Molecular targets of phytochemicals for cancer prevention” from *Natural reviews cancer* (IF: 53.03) was written by Lee KW, which indicated that cancer-related inflammation and immunity were the hot research topics in this field, which also provided insights for future research referring to the role of natural products in the prevention and treatment of cancer.

As shown in the timeline view of co-cited references, most studies were published after 2000. “Natural killer cell” (Cluster #0), “NK cell” (Cluster #3) and “natural killer t cell” (Cluster #9) with the warmest color and largest nodes, contained the most publications, indicating that the underlying mechanism of the natural products in cancer treatment may be through the regulation of natural killer cells, which was consistent with the role of natural products in cancer related inflammation and immunity mentioned in the above highly cited references. Cluster #1 (cinnamomum verum component) and Cluster #7 (oleanolic acid) demonstrated that they were the most well studied natural products in this field recently. Meanwhile, Cluster #4 (epigenetic regulation) and Cluster #10 (extracellular wesicle) were the most popular mechanism studies and directions of natural products in cancer research.

### Keyword Analysis

We used CiteSpace software to analyze the keywords and visualized the clusters, which mainly included terms related to mechanisms. For further analyses, based on the analysis by citation counts and centrality, the potential mechanisms of natural products as anti-cancer drugs *in vitro* may be mediated by the regulation of NF-kappa B signaling pathway (Qin et al., 2017), oxidative stress (Tan et al., 2018), or by the promotion of cell apoptosis (Zeng et al., 2019) and cytotoxicity (Lin et al., 2020). The keywords analysis also showed that flavonoid, curcumin polyphenol, and resveratrol were the most well studied natural products, while breast cancer, prostate cancer and colorectal cancer were the most widely studied cancer types in this field, which was similar to the results reported by Yeung et al. (2018). The similar results further confirmed that these natural products and cancer types were the most popular research directions in this field. Specifically, flavonoid and resveratrol, which exist in various dietary sources (vegetables and fruits) have been widely investigated in cancer research. Multiple studies have confirmed that flavonoid possessed vigorous anti-oxidant activity and also had anti-carcinogenic properties (Anwar et al., 2018; Dobrzynska et al., 2020; Moradi et al., 2020). Meanwhile, epidemiological studies have concluded that dietary flavonoid intake was associated with a reduced risk of various cancers, including breast cancer, prostate cancer, and colorectal cancer (Kim et al., 2017; Rodríguez-García et al., 2019; Feng et al., 2020). Sinha et al. (2016) summarized the anti-breast cancer mechanism of resveratrol and showed that it could prevent cancer cell proliferation, metastasis, inhibit epigenetic alterations, induce cell apoptosis and sensitize the drug-resistant cancers cells to chemotherapy. In addition, several studies reported that the mechanisms of polyphenols inducing cancer cell senescence and targeting tumor microenvironment to prevent and treat cancer (Bian et al., 2020; Cueva et al., 2020). Moreover, most of the papers about curcumin focused on its anti-inflammatory, anti-oxidation and anti-tumor activities (Farhood et al., 2019; Hayakawa et al., 2020).

The hotspots and development trends of natural products in cancer research can be revealed by the combined analysis of the bursty keywords and the cluster of keywords. It is known to all that the progress of technology promotes the development of the discipline. The keywords of “molecular docking” and “gold nanoparticle,” emerged from 2018, shown the strength of citation burst was 17.1336 and 12.2994, respectively, which meant that these technologies had been very popular in this field in the past 2 years. Molecular docking could effectively screen the binding sites of natural products and target genes. For instance, Wang et al. used molecular docking to confirm that two ginsenosides Rg5 and Rk1, with similar structure, were directly bound to Annexin A2. It suggested that Rg5 and Rk1 might be promising natural compounds for cancer treatment by targeting Annexin A2 (Wang et al., 2018). Nanotechnology-based antioxidants and therapeutic agents are viewed as next-generation tools for cancer treatment, and graphene has become a preferred nano-therapeutic template due to its advanced properties and cellular interaction mechanisms. Al-Ani et al. (2019) found that the hybrid nanocomposite curcumin-capped gold nanoparticle-reduced graphene oxide affected anti-oxidant potency and selective cancer cytotoxicity. Therefore, more in-depth research on natural products in cancer treatment by using molecular docking and gold nanoparticle is expected to be conducted in the future.

With the development of microbiome technology, an increasing number of studies suggested that gut microbiota played an important role in the process of cancer. Interestingly, our results also illustrated that the modulation of gut microbiota and the improvement of gut dysbiosis could be potential targets for cancer therapy for some natural products (Chen et al., 2020b; Cheung et al., 2020; Trošelj et al., 2020). Recent studies have indicated that the immune checkpoint blockade has gradually become a new direction in the anti-tumor research (Patel and Minn, 2018). A study has concluded that resveratrol was capable to suppress anti-tumor immunity by controlling mainly PD-L1 expression (Yang et al., 2021). Therefore, targeting gut microbiota and immune checkpoint is a promising strategy in cancer therapy. As the burst keywords and visualization of the cluster demonstrated that the topics of “gut microbiota,” “blockade” and “immune checkpoint” were noted as new research hotspots.

Additionally, the natural products, including leaf extract, rosmarinic acid, oleanolic acid and cinnamomum verum component, are currently the research hotspots in this field. Research hotspots related to these drugs have focused on the mechanisms of anti-cancer, including anti-inflammatory (Rodríguez-Luna et al., 2019), protection against DNA damage (Boss et al., 2016), anti-metastatic (Syed Najmuddin et al., 2016), regulation signaling pathways (such as NF-κB, COX-2, and STAT signaling) (Perng et al., 2016; Song et al., 2017; Narożna et al., 2020), suppression of cell migration and modulation of lncRNA expression (Zhang et al., 2018), etc. Thus, these natural products may become potential clinical anti-tumor drugs in the future.

### Limitations

Some limitations should be illustrated in our study. On the one hand, the data were extracted only from the Web of Science core collection database, and articles published in other sources such as PubMed and Scopus might be missed. On the other hand, only English articles were included from the database, which may possibly lead to source bias.

## Conclusion

To our knowledge, our study is the first comprehensive bibliometric analysis of natural products in cancer research. Increasing evidence hasindicated the role of natural products in cancer diseases. In this study, the results showed that the United States has carried out research earlier and maintained a steady growth trend, indicating it has made a great contribution in this field. Since 2016, the number of annual publications in China surpassed that in the United States, which may be related to the government’s encouragement and financial support for scientific research. Moreover, the increasing number of annual publications also suggested that the value of natural products in cancer treatment has attracted more and more attention all over the world.

From the perspective of cooperative relations, institutional cooperation is an aggregative trend. However, there was a lack of cooperation among institutions from different countries/regions, which indicated that more institutional collaboration is needed. Notably, the Chinese Academy of Sciences and the National Institutes of Health (United States) played an important role in the research of natural products in cancer. Thus, institutions can cooperate closely with them to promote the development of the field in the future.

The most productive journal and the most frequently co-cited journal was *Molecular* and *Cancer Research*. Additionally, the most productive author and co-cited authors were Efferth Thomas and Newman DJ, respectively. Therefore, researchers can better grasp the relevant research progress in this field by consulting the articles published by these journals or authors. Meanwhile, these productive authors also will be potential collaborators in this field.

Importantly, our results also showed that molecular docking and gold nanoparticle may be the most advanced and popular technologies in recent research. In addition, the natural products, including leaf extract, rosmarinic acid, oleanolic acid, and cinnamomum verum component may be the most popular ones, and may become potential clinical anti-tumor drugs in the future. Most studies have focused on basic mechanism outcomes, such as the regulation of natural killer cell, promotion of apoptosis, and the reduction in oxidative stress. The underlying anti-cancer effect of natural products, especially those related to gut microbiota and immune checkpoint, may soon become research hotspots and should be closely monitored. Notably, few studies have reported clinical trials of natural products in cancer research. Thus, more in-depth research on the therapeutic effects and safety examination of natural products on cancer patients is needed.

In summary, this research has firstly indicated a comprehensive knowledge map for the natural products on cancer research, which provided potential collaborators and institutions, and hot topics. Furthermore, it also offered a perspective to the developing trend, which may help researchers explore new directions for future research in this field.

## Data Availability

The raw data supporting the conclusion of this article will be made available by the authors, without undue reservation.
